# Evaluating dexmedetomidine in mitigating emergence agitation and perioperative complications in pediatric tonsillectomy and/or adenoidectomy: a systematic review and meta-analysis

**DOI:** 10.3389/fphar.2025.1681936

**Published:** 2025-10-29

**Authors:** Jihong He, Xianghong Lian, Ting Luo

**Affiliations:** ^1^ Department of Neurosurgery, The 3rd Affiliated Hospital of Chengdu Medical College, Pidu District People’s Hospital, Chengdu, China; ^2^ Department of Pharmacy, West China Second University Hospital, Sichuan University, Chengdu, China; ^3^ Evidence-Based Pharmacy Center, West China Second University Hospital, Sichuan University, Chengdu, China; ^4^ Key Laboratory of Birth Defects and Related Diseases of Women and Children, Sichuan University, Ministry of Education, Chengdu, China

**Keywords:** dexmedetomidine, pediatric, tonsillectomy, adenoidectomy, perioperative complications, emergence agitation, meta-analysis

## Abstract

**Background:**

Perioperative complications and emergence agitation (EA) are common after pediatric tonsillectomy and/or adenoidectomy (T&A), and may be influenced by the use of preoperative sedatives. The effectiveness of dexmedetomidine (Dex) in minimizing these risks is still debated.

**Methods:**

We searched EMBASE, PubMed, and the Cochrane Library for randomized controlled trials (RCTs) assessing the safety and effectiveness of Dex in pediatric T&A, with comparisons made against placebo and/or alternative comparators. The search included studies published before March 2025. Retrieved data included the incidence of EA, the percentage (%) of cases requiring rescue analgesics, and perioperative complications, such as hypotension and bradycardia, and perioperative respiratory adverse events (PRAEs). The meta-analysis was performed using RevMan 5.3.

**Results:**

Thirty-six RCTs including 3,773 children were included. Compared with placebo, benzodiazepines, and opioids, Dex significantly reduced the occurrence of EA [OR = 0.23, 95% CI (0.16, 0.32), I^2^ = 44%] [OR = 0.51, 95% CI (0.28, 0.93), I^2^ = 44%] [OR = 0.19, 95% CI (0.09, 0.39), I^2^ = 0%] (P < 0.05). Subgroup analysis of delivery methods, timing, and dosage (Dex ≥0.5 μg/kg) indicated that Dex significantly decreased the incidence of EA (P < 0.05). Furthermore, compared with placebo and benzodiazepines, Dex markedly decreased the incidence of patients necessitating rescue analgesia, while no statistically significant difference was noted versus opioids. Dex also significantly decreased the incidence of PRAEs (oxygen saturation (%) and laryngospasm) [OR = 0.41, 95% CI (0.25, 0.69), I^2^ = 0%] [OR = 0.38, 95% CI (0.19, 0.78), I^2^ = 0%] (P < 0.05) However, there was no significant difference in the incidence of hypotension or bradycardia [OR = 2.28, 95% CI (0.99, 5.23), I^2^ = 0%, P = 0.05] [OR = 2.00, 95% CI (1.00, 3.98), I^2^ = 2%, P = 0.05]. Finally, recovery time did not differ significantly between the Dex and control groups.

**Conclusion:**

Dex may mitigate EA and perioperative complications while enhancing recovery quality following T&A in pediatric patients.

## 1 Introduction

Tonsillectomy, with or without adenoidectomy (T&A), is a routinely performed operation in children under general anesthesia (Hall et al., 2017; Cho et al., 2018). Surgical procedures may result in throat irritation and considerable stress response, potentially linked to notable perioperative complications in 9.4% of cases, including emergence agitation (EA), perioperative complications (such as perioperative respiratory adverse events (PRAEs), nausea or vomiting, and severe pain) (Belyea et al., 2014). Despite their short duration, these occurrences may heighten the risk of self-harm, extend the stay in the PACU, demand more intensive nursing support, and increase healthcare expenditures (Zh et al., 2021). Effective perioperative management may reduce these complications, and numerous medications administered preoperatively or intraoperatively, such as dexmedetomidine (Dex), propofol, midazolam, opioids, ketofol, and ketamine, have been studied for their efficacy in preventing EA and perioperative complications in children (Urits et al., 2020). Nonetheless, considerable discrepancies in management practices persist (Steward et al., 2011).

Dex, characterized by its selectivity for α2-adrenoreceptors, exhibits multiple pharmacologic actions—sedation, analgesia, anesthesia, and sympatholysis—combined with vasoconstriction and minimal respiratory suppression, making it a valuable sedative-analgesic agent for children undergoing T&A under anesthesia (Mahmoud and Mason, 2015). The effectiveness of Dex in this setting has been documented in several clinical trials, that have employed various delivery methods and doses (Pestieau et al., 2011a; Li LQ. et al., 2018). Its role in mitigating EA has been the subject of numerous systematic reviews and meta-analyses (Cho et al., 2018; He et al., 2013). Nevertheless, current evaluations have not specifically addressed pediatric T&A. Previous meta-analyses predominantly contrasted Dex with opioids (e.g., morphine and fentanyl) in tonsillectomy operations (Cho et al., 2018; He et al., 2013; Rao et al., 2020); however, and their findings were limited by small sample sizes, significant heterogeneity, or the inclusion of nonrandomized trials. These comprehensive studies failed to account for the manner of delivery (continuous injection versus intranasal), the comparative target (placebo versus opioid), varying dosages, or PRAEs. Given the limited availability of recent randomized controlled trials (RCTs), the therapeutic profile of Dex in juvenile T&A has not yet been comprehensively reviewed. To address this gap, we incorporated trials utilizing delivery routes [intravenous (IV), intranasal, and oral] and varied timing of Dex administration (premedication, post-anesthesia induction, and prior to surgical closure) across low (<0.5 μg/kg), moderate (≥0.5 to <1 μg/kg), and high (≥1 μg/kg) dosing groups. The current meta-analysis is designed to evaluat the effects of Dex on various administration methods and dosages of Dex to enhance patient experience immediately following T&A, thereby providing evidence for healthcare professionals and pharmaceutical research and development.

## 2 Materials and methods

In conducting this meta-analysis, we complied with the Preferred Reporting Items for Systematic Reviews and Meta-Analyses (PRISMA) criteria and applied procedures specified in the Cochrane Handbook (Higgins and Green, 2011).

### 2.1 Search methodology

Our search was conducted in the PubMed, Embase, and Cochrane Library databases for articles published before March 2025. Additional studies were identified through three clinical trial registry platforms: Clinical Trials.gov, the WHO Clinical Trials Registry Platform, and the Cochrane Central Registry of Controlled Trials. The search strategy was specific for each database and included a combination of medical subject headings and free-text terms (“Dex” or “Precedex”), pediatric populations, and tonsillectomy procedures.

### 2.2 Eligibility criteria

We included studies that (1) involved patients aged 0–18 years necessitating T&A procedures, classified as American Society of Anesthesiologists (ASA) I–III; (2) evaluated Dex against placebo and/or active comparators in pediatric T&A, with no restrictions on the route of administration; (3) placed no restrictions on the control group composition; (4) reported the frequency of EA and perioperative complications (e.g., nausea, vomiting, cough, laryngospasm, hypotension, bradycardia) as primary outcomes and the frequency of subjects requiring rescue analgesics and recovery time as secondary outcomes; and (5) were RCTs. We excluded studies that (1) involved intensive care unit patients; (2) included adults; (3) lacked extractable data; (4) were review articles, letters, or animal studies, or lacked a comparator; and (5) were duplicates of previously published work.

### 2.3 Data extraction

Two investigators separately retrieved data utilizing a preestablished extraction template. The information gathered included the study author, publication year, sample size, average age, intervention measure, dosage, surgical procedure, and relevant outcomes *as per* the inclusion criteria.

Two investigators also independently evaluated all titles and abstracts to select studies for full-text screening. Eligibility criteria were subsequently applied independently for final inclusion. Conflicts over article eligibility were addressed through deliberation, during which the reviewers articulated their reasoning and reached mutual agreement on inclusion or exclusion. If disagreements persisted, a third reviewer adjudicated the final inclusion decision.

### 2.4 Evaluation of bias risk

A bias assessment was conducted for the selected RCTs using the Cochrane risk-of-bias (RoB) tool (Higgins and Green, 2011).

### 2.5 Statistical analysis

The pooled analysis was implemented by use of Review Manager 5.3, and effect measures were calculated as either odds ratios (ORs) or standardized mean differences (SMDs), with 95% confidence intervals (95% CIs) provided.

We quantified heterogeneity by computing the I-squared (I^2^) value, and a fixed-effects model was employed. An I^2^ value greater than 50% was deemed indicative of significant heterogeneity; in such cases, contributing factors were explored, and a random-effects model was adopted as needed.

Furthermore, to examine the impact of Dex on EA occurrence, subgroup analyses were performed *as per* prior hypotheses from three aspects: varying administration routes (IV versus intranasal), differing administration times (post-induction of anesthesia, pre-surgery conclusion), and dosage variations (low (<0.5 μg/kg), moderate (≥0.5 to <1 μg/kg)), and high doses (≥1 μg/kg)]. We established six distinct subgroups according to several event types: vomiting, cough, hypotension, bradycardia, oxygen saturation (%), and laryngospasm.

## 3 Results

### 3.1 Literature search and study profile

**FIGURE 1:**
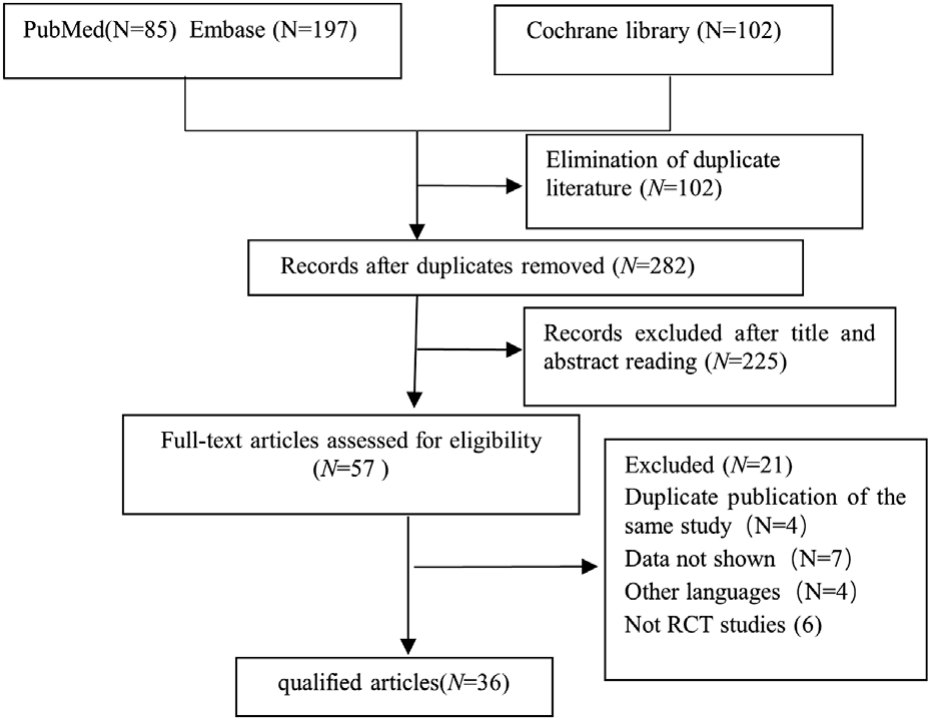
Study retrieval and selection workflow.

From an initial pool of 384 screened articles, 36 relevant studies published from 2005 to 2024 were incorporated into this meta-analysis (Figure 1). a total of 3,773 children participated in this research. Dex was administered at 0.three to four μg/kg, which aligns with dosage guidelines for pediatric sedation during noninvasive operations and reflects contemporary clinical use (Mace et al., 2008; Aldamluji et al., 2021) (Table 1).

**TABLE 1:** Characteristics of included randomized-controlled trial.

Number	Study ID	Intervention	Sample size	Age (years)	Weight (kg)	Anesthesia	Time	Surgery	ASA	Recovery time (min)	EA (%)	Rescue analgesic frequency (%)	Adverse events
1	Cao et al. (2016)	DEX	30	4.1 ± 1.5	20.9 ± 5.8	intravenous (IV) dexmedetomidine 1ug/kg over 10 min, followed by 0.5ug/kg/h continuous infusion	until to 5 min before the end of surgery	tonsillectomy with or without adenoidectomy	ASA I or II	15.2 ± 5.1∗	NA	NA	NA
		Group control	30	3.9 ± 1.8	21.7 ± 7.9	the same volume of 0.9% saline				12.4 ± 3.5			
2	Ali and Abdellatif (2013)	The control group (Group C)	40	3.9 ± 1.6	18.7 ± 4.5	received 10 mL NaCI 0.9%	About 5 min before the end of surgery	adenotonsillectomy	ASA I-II	NA	12.5	NA	②
		propofol group (Group P)	40	4.2 ± 1.4	19.8 ± 4.6	propofol 1 mg/kg					10		
		Dexmedetomidine group (Group D)	40	4.3 ± 1.3	19.5 ± 4.8	IV dexmedetomidine 0.3 ug/kg diluted in 10 mL NaCI 0.9%					12.5		
3	Tsiotou et al. (2018)	Dexmedetomidine group (A)	31	6.1 (2.6)	22.8 (9.5)	IV dexmedetomidine 1 ug/kg	After the induction of anesthesia	tonsillectomy with and without adenoidectomy	ASA I or II	NA	4	NA	①
		Group control (B)	29	6.3 (2.6)	24.03 (10.9)	normal saline solution					12 (41.4)		
4	Soliman and Alshehri (2015)	Group control (A)	75	8.38 ± 3.00	21.12 ± 5.53	the patients received sevoflurane 1%–3% during the surgery	after induction of anesthesia	adenotonsillectomy	ASA I–II	NA	29	NA	②⑤⑥
		Dexmedetomidine group (B)	75	8.56 ± 3.08	21.48 ± 3.99	IV dexmedetomidine 0.5 ug/kg					6 (8%)		
5	Bai et al. (2016)	Dexmedetomidine group	62	9.8 ± 2.9	33.6 ± 11.0	dexmedetomidine 0.5 μg/kg, intravenous	After stable anesthesia	Tonsillectomies	ASA I–II	NA	5	NA	①②⑤⑥
		T group	62	9.7 ± 3.3	33.5 ± 9.8	the same amount of normal saline					15		
6	Mizrak et al. (2013)	Group D	30	8.7 ± 3.6	28.0 ± 16.6	dexmedetomidine 0.5 mg/kg	10 min before the induction of anesthesia	undergoing adenotonsillectomy	ASAI or II.	6.90 ± 2.92	NA	NA	NA
		Group C	30	9.8 ± 4.0	23.9 ± 10.2	placebo bolus				6.0 ± 2.94			
7	Li et al. (2018b)	group D	30	5.1 6 ± 1.15	22.6 ± 7.09	infused 0.2 ug/kg/hour dexmedetomidine	until the end of the surgery	undergoing tonsillectomy	ASAI or II	36.70 ± 10.70	NA	NA	②
		group C	40	5.5 6 ± 1.17	22.4 ± 6.68	placebo bolus				40.68 ± 11.95			
8	El-Hamid and Yassin (2017)	Group D	43	4.4 ± 1.3	17.4 ± 3.4	intranasal dexmedetomidine at 1 μg/kg	after the induction of general anesthesia	tonsillectomy and/or adenoidectomy	ASA I and II	35.93 ± 10.21	6.98%	NA	②
		Group C	43	4.2 ± 0.93	18.6 ± 4.1	received intranasal saline 0.9%				39.17 ± 9.86	58%		
9	Li et al. (2018c)	D1 groups	30	4.47 ± 1.17	19.82 ± 5.51	intranasally dexmedetomidine 1ug/kg	25–40 min before surgery	adenoidectomy with or without tonsillectomy	ASA I and II	NA	43.30%	NA	②
		D2 groups	30	4.53 ± 1.55	20.05 ± 5.79	intranasally dexmedetomidine 2 ug/kg					30.00%		
		S groups	30	4.37 ± 1.30	18.67 ± 4.10	saline of the same volume					63.30%		
10	Wang et al. (2013)	group D1	20	4.2 ± 0.8	19.0 ± 3.7	intranasal Dexmedetomidine 1 ug/kg	30 min before anesthesia induction	adenotonsillectomy	ASA I or II	NA	NA	NA	NA
		group D2	20	4.3 ± 1.1	18.9 ± 3.7	intranasal Dexmedetomidine 2 ug/kg							
11	Yi et al. (2022)	dexmedetomidine 0.5 group	58	6.06 ± 1.71	23.28 ± 7.42	dexmedetomidine 0.5 μg/kg	After intubation	adenotonsillectomy	ASA I or II	66.67 ± 16.12	NA	NA	④
		dexmedetomidine 1 group	62	6.17 ± 1.80	23.03 ± 6.74	dexmedetomidine 1 μg/kg				52.38 ± 15.33			
12	Shafa et al. (2021)	dexmedetomidine 1ug/kg group	35	6.5 ± 2.0	21.9 ± 6.8	dexmedetomidine 1ug/kg	before the beginning of the operations	denotonsillectomy	ASA I or II	48.8 ± 6.6	NA	NA	⑥
		dexmedetomidine 2ug/kg group	35	6.6 ± 2.01	21.6 ± 5.4	dexmedetomidine 2ug/kg				51.4 ± 7.5			
		Placebo group	5	6.0 ± 2.1	21.7 ± 7.3	saline of the same volume				54.4 ± 7.3			
13	Abo Elfadl et al. (2022)	Group L	45	5.2 ± 1.3	20.32 ± 4.98	levobupivacaine 0.25%	before the beginning of the operation	tonsillectomy with or without adenoidectomy	ASA I-II	10.2 ± 1.67	NA	NA	②③⑤⑥
		Group LD	45	5.1 ± 1.3	19.65 ± 4.41	levobupivacaine plus dexmedetomidine 1 μg/kg				10.8 ± 1.37			
14	Guler et al. (2005)	Dexmedetomidine group	30	4.7 ± 1.2	18.43 ± 3.47	Dexmedetomidine (0.5ug/kg)	About 5 min before the end of surgery	adenotonsillectomy	ASA I	9.30 ± 2.9*	5* (17)	7* (23)	①②
		Placebo group	30	4.5 ± 1.2	17.46 ± 4.09	the same volume of sodium chloride				7.20 ± 2.7	17 (57)	16 (53)	
15	Abdel-Ghaffar et al. (2019)	Group C	30	5 (2.5–6)	15 (12–22)	saline placebo	preoperative premedication	tonsillectomy	ASA I-II	NA	NA	NA	①
		Group dexmedetomidine I	30	5 (3–6)	15 (10–25)	buccal trans-mucosal dexmedetomidine 0.5 ug/kg							
		Group dexmedetomidine II	30	5 (3–6)	18 (10–25)	buccal trans-mucosal dexmedetomidine 1 ug./kg							
16	Hao et al. (2020)	RL	56	6.0 (2.1)	19.6 (3.1)	0.25% ropivacaine and 1 μg/kg dexmedetomidine	After intubation	The tonsillectomy and adenoidectomy	ASA I-II	NA	NA	NA	NA
		R	59	5.7 (2.0)	21.0 (3.9)	0.25% ropivacaine							
17	Yao et al. (2022)	Control	30	4.3 ± 1.1	19.9 ± 4.5	placebo	before induction	tonsillectomy and/or adenoidectomy	ASA I	37.23 ± 7.71	NA	NA	NA
		PPIA group	30	4.6 ± 1.2	20.9 ± 4.5	a parent				40.20 ± 7.28			
		Dexmedetomidine group	30	4.4 ± 1.2	18.4 ± 4.9	intranasal dexmedetomidine 1.0 μg/kg				40.37 ± 7.61			
		PPIA + Dexmedetomidine group	30	4.6 ± 1.4	19.7 ± 5.3	intranasal dexmedetomidine 1.0 μg/kg+ a parent’s arms				42.23 ± 6.78			
18	Di et al. (2018)	Group D0	25	5.3 ± 1.3	19.5 ± 3.4	saline infusion	over 10 min in pre-op area	tonsillectomy	ASA I-II	NA	NA	NA	②
		Group D1	26	5.0 ± 1.1	19.6 ± 3.7	dexmedetomidine 1 μg/kg infusion							
		Group D2	24	5.1 ± 1.0	19.8 ± 3.4	dexmedetomidine 2 μg/kg infusion							
19	Golmohammadi et al. (2024)	Intervention group	38	3.97 ± 1.04	13.88 ± 1.39	an infusion of 0.5 μg/kg/h of dexmedetomidine	after induction of anesthesia	adenoidectomy	ASA I	9.65 ± 5.14	34.21%	NA	②④⑤⑥
		Control group	38	3.62 ± 1.12	14.55 ± 1.5	an equal volume of normal saline infusion				7.31 ± 2.44	53.95%		
20	Hadi et al. (2015)	KETODEX	45	4.22 ± 1.32	18.52 ± 4.60	dexmedetomidine 0.3 ug/kg i.v	About 10 min before the end of surgery	adenotonsillectomy	ASA I–II	NA	11%	NA	③④
		Control	47	4.22 ± 1.12	18.37 ± 5.21	volume-matched normal saline					47%		
21	Shahhosseini et al. (2023)	A	25	9 ± 2	NA	infused in dose of 0.6 μg/kg	After induction	tonsillectomy	ASA I–II	50 ± 9	NA	NA	NA
		B	25	9 ± 2	NA	infused in dose of 0.3 μg/kg				67 ± 8			
		C	25	9 ± 2	NA	normal bolus saline				75 ± 7			
22	Zhang et al. (2022)	Control	20	4.53 ± 1.32	21.35 ± 9.69	normal saline	from the induction	adenoidectomy and tonsillectomy	ASA I or II	14.95 ± 3.57	10 (50%)	15	①②③⑥
		Dexmedetomidine	20	4.81 ± 1.09	21.60 ± 5.12	intravenously 0.4 μg/kg dexmedetomidine				14.86 ± 3.89	5 (25%)	7	
		Dexmedetomidine + Alf1	20	5.13 ± 1.29	23.15 ± 9.31	intravenously with 0.4 μg/kg dexmedetomidine and alfentanil (10 μg/kg)				15.61 ± 4.59	1 (5%) *	3	
		Dexmedetomidine + Alf2	20	5.11 ± 1.23	22.69 ± 9.83	intravenously with 0.4 μg/kg dexmedetomidine and alfentanil (20 μg/kg)				19.25 ± 4.38	0 (0) *	2	
23	Abdel-ghaffar and Abdel-Haleem (2011)	Placebo group	28	8.92 ± 2.53	30.60 ± 6.61	50 mL saline 0.9% iv	after intubation 3– 5 min before start of surgery	Adenoidectomy/tonsillectomy	ASA I---II	NA	NA	NA	NA
		Dexmedetomidine IV	28	8.26 ± 2.35	28.85 ± 8.35	1ug/kg dexmedetomidine given by iv. infusion							
		dexmedetomidine.PT	28	8.60 ± 2.31	30.28 ± 8.70	l ug/kg dexmedetomidine							
24	Shen et al. (2022)	Normal saline	125	12 (9.6)	17.2 (15.4–19.1)	1 mL of 0.9%saline	anesthesia induction 前	Tonsillectomy/Adenoidectomy	ASA I or II	15.0 (12.0–17.0)	27 (21.6)	23 (18.4)	①②③
		Midazolam	124	17 (13.7)	15.9 (14.6–18.3)	intranasal midazolam (0.1 mg/kg)				14.0 (12.0–16.0)	36 (29.0)	30 (24.2)	
		Dexmedetomidine	124	16 (12.9)	16.3 (14.6–18.4)	intranasal Dexmedetomidine 2.0 μg/kg				15.0 (12.0–17.0)	12 (9.7)	14 (11.3)	
25	Cho et al. (2019)	Midazolam	32	7.2 ± 2.2	28.9 ± 11.3	0.03 mg/kg midazolam, IV	Five minutes before the end of surgery	elective tonsillectomy	ASA I or II	19.0 [13.0–23.0]	10 (31.3%)	5 (15.6%)	②
		Dexmedetomidine	34	6.7 ± 2.4	26.3 ± 10.0	Dexmedetomidine 0.3 μg/kg, IV				18.5 [15.0–25.0]	9 (26.5%)	3 (8.8%)	
26	Mahfouz et al. (2011)	Group D	60	8.2 ± 1.4	18.40 ± 4.74	intranasal 1 ug/kg dexmedetomidine	before induction of anesthesia	adenotonsillectomy	ASA I	38.27 ± 4.31	NA	8 (13)	NA
		Group M	60	8.1 ± 2.3	17.9 ± 5.89	10 mL apple juice orally as a placebo				36.77 ± 4.62		15 (25)*	
27	Akin et al. (2012)	Midazolam	45	6 (2–9)	19.5 (11–35)	0.2 mg/kg of intranasal midazolam	before the induction of anesthesia	adenotonsillectomy	ASA I	NA	NA	15 (33.3)	②③
		Dexmedetomidine	45	5 (3–9)	18.5 (11–35)	intranasal 1 ug/kg dexmedetomidine						6 (13.3)	
28	Elagamy et al. (2020)	Group (Dexmedetomidine)	80	4.5 ± 0.81	17.18 ± 2.5	0.5 μg/kg Dexmedetomidine by IV infusion	over 10 min after induction of anesthesia	adenotonsillectomy	ASA I or II	40.38 ± 7.43	NA	NA	②⑤⑥
		Group (Nal)	80	4.7 ± 1.2	16 ± 2.53	0.9% normal saline IV				37.16 ± 9.38			
29	Zhuang et al. (2011)	Morphine	30	5.0 (2.5)	21.9 (9.4)	Intravenous dexmedetomidine 1 ug/kg	anaesthetic induction	adenotonsillectomy	ASA I or II	NA	NA	30%	②
		Dexmedetomidine	30	4.5 (1.7)	22.6 (7.9)	Intravenous dmorphine 100 ug/kg						57%	
30	Bedirli et al. (2017a)	Group T	39	8.4 ± 2.1	28.3 ± 3.7	2 mg/kg tramadol	After intubation	Adenotonsillectomy	ASA I–II	15.2 ± 4.7	NA	19	②
		Group D	38	6.7 ± 3.1	27.1 ± 2.7	1 μg/kg dexmedetomidine				37.6 ± 5.4		17	
31	Koceroglu et al. (2019)	Dexmedetomidine group	30	6.17 ± 2.07	22.77 ± 5.94	1 μg/kg dexmedetomidine	tthe end of surgery	adenotonsillectomies	ASA I -II	NA	NA	NA	②④
		Tramadol group	30	5.4 ± 2.19	19.6 ± 7.24	1.5 mg/kg tramadol							
32	Modir et al. (2024)	Dexmedetomidine -ropivacaine	54	6.97 ± 1.45	NA	0.25% ropivacaine +1 μg/kg dexmedetomidine	before surgical incision	tonsillectomy	ASA I -II	NA	NA	7 (21.21)	②
		Tramadol-ropivacaine	54	6.97 ± 1.45	NA	the same ropivacaine solution +2 mg/kg tramadol						29 (87.87)	
		Placebo-ropivacaine	54	6.97 ± 1.45	NA	the same solution + normal saline						33 (100)	
33	Patel et al. (2010)	Group D (Dexmedetomidine)	61	4.2 ± 2.1	18.3 ± 5.7	IV dexmedetomidine 2 μg/kg	5 min before the end of the surgery)	Tonsillectomy and Adenoidectomy	ASA II–III	7.18 ± 4.05	NA	22 (36.1)	④
		Group F (fentanyl)	61	3.8 ± 1.5	20.4 ± 8.6	IV fentanyl bolus 1 μg/kg				8.75 ± 4.06	NA	6 (9.8)	
		Dexmedetomidine	20	4.81 ± 1.09	21.60 ± 5.12	intravenously 0.4 μg/kg dexmedetomidine				14.86 ± 3.89	25.00%		
		Dexmedetomidine + Alf1 group	20	5.13 ± 1.29	23.15 ± 9.31	intravenously with 0.4 μg/kg dexmedetomidine and alfentanil (10 μg/kg)				15.61 ± 4.59	5.00%		
		Dexmedetomidine + Alf2 group	20	5.11 ± 1.23	22.69 ± 9.83	intravenously with 0.4 μg/kg dex and alfentanil (20 μg/kg)				19.25 ± 4.38	0.00%		
34	Pestieau et al. (2011b)	fentanyl 1 μg/kg (Group 1)	26	4 (2–9.9)	17.4 (4.0)	fentanyl 1 μg/kg	immediately after endotracheal intubation	Tonsillectomy with or without adenoidectomy	ASA I or II	NA	15 (58)	25 (96)	NA
		fentanyl 2 μg/kg (Group 2)	25	4.7 (2.1–11.8)	16.6 (2.8)	fentanyl 2 μg/kg					14 (56)	18 (72)	
		Dex 2 μg/kg (Group 3)	25	5.3 (2.3–12.9)	17.1 (3.4)	dexmedetomidine 2 μg/kg					5 (20)	7 (28)	
		Dex 4 μg/kg (Group 4)	25	4.3 (2.2–11.9)	18.1 (3.5)	dexmedetomidine 4 μg/kg					4 (16)	7 (28)	
35	Anjana et al. (2021)	Group F (fentanyl)	60	7	24 (11)	fentanyl 2 μg/kg intravenously	premedication	tonsillectomy	ASA I or II	14 (6.5)	NA	NA	①
		Group D (Dex)	60	7.5	24 (12)	dexmedetomidine 0.5 μg/kg as intravenous infusion				13 (4)			
36	Erdil et al. (2009)	Group C	30	4.2 ± 1.3	17.3 ± 4.0	saline solution	After induction	adenoidectomy with or without bilateral myringotomy	ASA I	12.0 ± 4.2	47% (14/30)	13	②
		Group F (fentanyl)	30	4.6 ± 1.4	17.0 ± 3.6	fentanyl 2.5 μg/kg				16.1 ± 5.3	13% (4/30)	4	
		Group D (Dexmedetomidine)	30	4.7 ± 1.4	17.9 ± 3.2	dexmedetomidine 0.5 μg/kg				12.7 ± 3.2	17% (5/30)	5	

① Cough; ② Nausea and vomiting; ③ Laryngospasm; ④ Low oxygen saturation; ⑤ Hypotension; ⑥ Bradycardia.

### 3.2 Quality assessment (RoB tool)

The Cochrane RoB tool assessed allocation concealment, random sequence generation, participant and personnel outcome assessment blinding, selective reporting, insufficient outcome data, and additional biases. Two reviewers, Xianghong Lian and Ting Luo, engaged in the process, and when conflicts arose between them, they deliberated, discovered the underlying causes, and then reached a final judgment. If an agreement could not be reached, the ultimate decision was rendered by a third evaluator.

**FIGURE 2:**
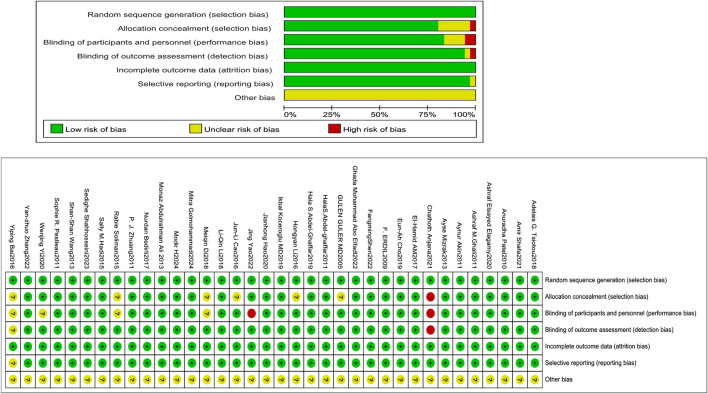
Evaluation of the quality of the included studies.

All investigations (36/36) employed an appropriate approach, using either manual or computerized random number tables. Of these, 29 explicitly addressed allocation concealment. Blinding of participants and research staff was implemented in 83.33% of the trials (30 out of 36). All trials (36/36) provided complete outcome data, and 97.22% of studies (35 out of 36) indicated no selective reporting upon review procedures. Blinding of outcome evaluation was conducted in 94.44% of trials (34 out of 36). Assessment of other biases was inconclusive in most trials (Figure 2).

### 3.3 Data examination

#### 3.3.1 Incidence of EA

**FIGURE 3:**
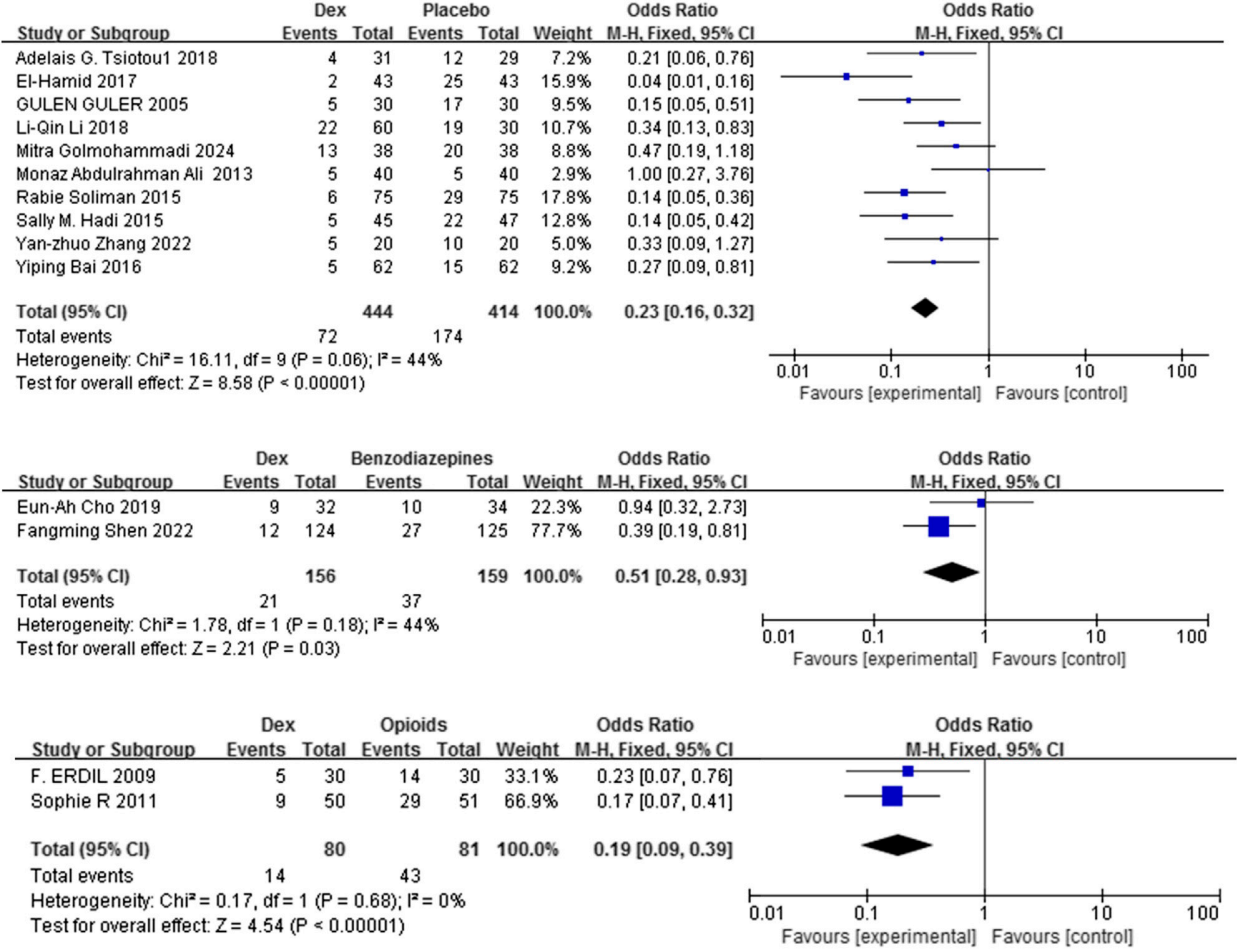
Forest plot illustrating EA incidence: Dex group versus control group.

Fourteen studies (Golmohammadi et al., 2024; Shen et al., 2022; Ali and Abdellatif, 2013; Tsiotou et al., 2018; Soliman and Alshehri, 2015; Bai et al., 2016; El-Hamid and Yassin, 2017; Li L-Q. et al., 2018; Guler et al., 2005; Hadi et al., 2015; Zhang et al., 2022; Cho et al., 2019; Pestieau et al., 2011b; Erdil et al., 2009) with 1334 patients evaluated the efficacy of Dex relative to that of three comparators in mitigating the risk of EA in children. Dex significantly reduced the incidence of EA compared with placebo, benzodiazepines, and opioids [OR = 0.23, 95% CI (0.16, 0.32), I^2^ = 44% [OR = 0.51, 95% CI (0.28, 0.93), I^2^ = 44%] [OR = 0.19, 95% CI (0.09, 0.39), I^2^ = 0%] (P < 0.0001) (Figure 3). No differences in significance levels emerged from the sensitivity analyses performed for each comparison.

#### 3.3.2 Frequency of rescue analgesic use

**FIGURE 4:**
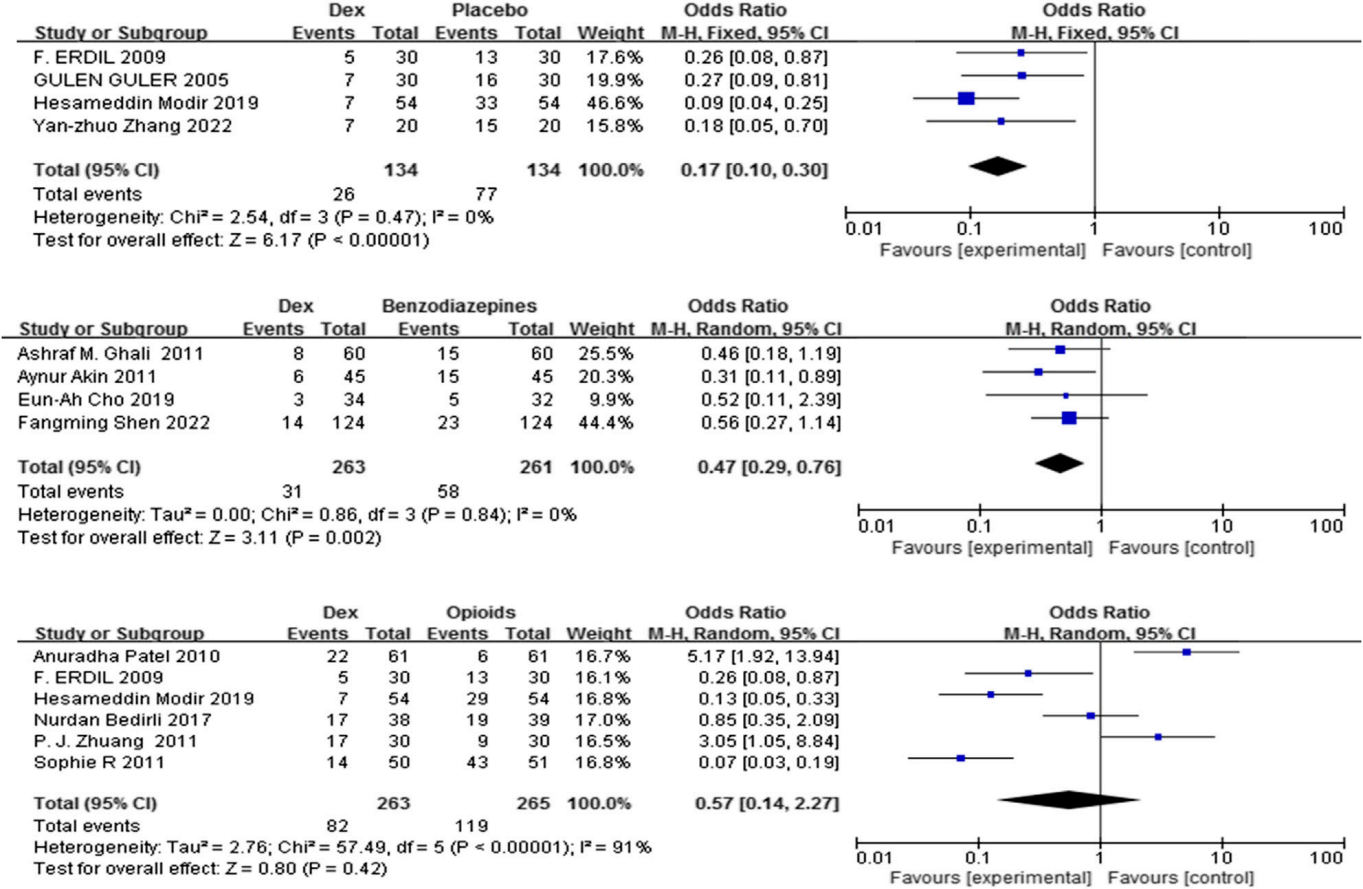
Forest plot comparing Dex and control groups on the frequency of patients who needed rescue analgesia.

Eleven trials (Shen et al., 2022; Guler et al., 2005; Zhang et al., 2022; Mahfouz et al., 2011; Akin et al., 2012; Zhuang et al., 2011; Bedirli et al., 2017a; Modir et al., 2024; Patel et al., 2010; Pestieau et al., 2011b; Erdil et al., 2009) including 1320 patients compared Dex with control (placebo, benzodiazepines, and opioids) on the frequency of rescue analgesic use. Dex substantially reduced the incidence of rescue analgesics compared with placebo, and benzodiazepines [OR = 0.17, 95% CI (0.10, 0.30), I^2^ = 0%,] [OR = 0.47, 95% CI (0.29, 0.76), I^2^ = 0%] (P < 0.0001) (Figure 4).

In contrast, no significant difference was found in the frequency of rescue analgesic use (%) between the Dex and opioid groups [OR = 0.57, 95% CI (0.14, 2.27), I^2^ = 91%, P = 0.42] (Figure 4).

#### 3.3.3 Recovery duration

**FIGURE 5:**
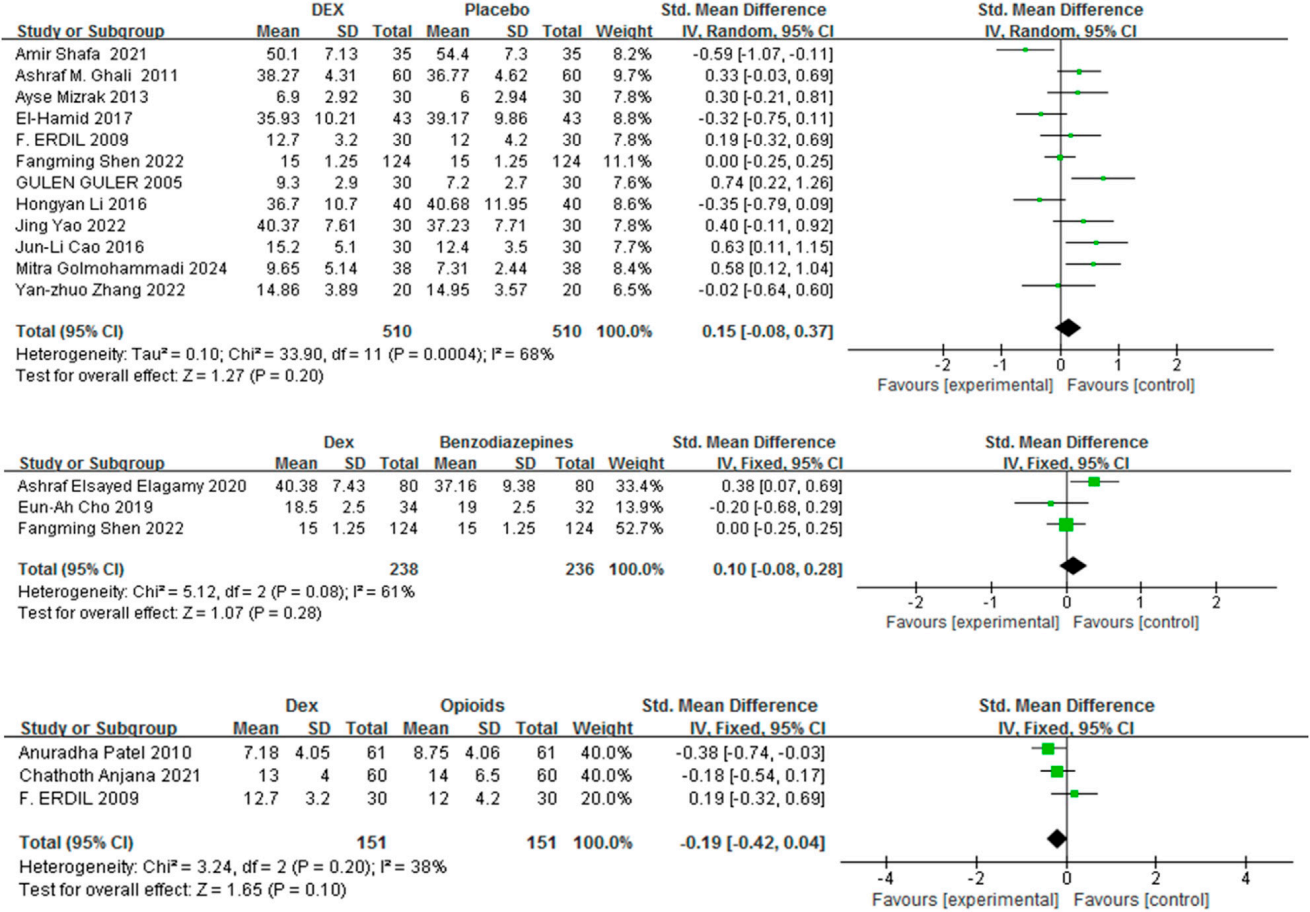
Forest plot comparing recovery time between Dex and control groups.

Recovery duration was defined as the period between the cessation of anesthesia and the patient’s eyes openings upon a verbal command. Fifteen studies (Golmohammadi et al., 2024; Shen et al., 2022; Shafa et al., 2021; Cao et al., 2016; Mizrak et al., 2013; Li H. et al., 2018; El-Hamid and Yassin, 2017; Guler et al., 2005; Zhang et al., 2022; Cho et al., 2019; Mahfouz et al., 2011; Elagamy et al., 2020; Patel et al., 2010; Anjana et al., 2021; Erdil et al., 2009) with 1320 patients were included, and the impact of Dex relative to a control group on recovery duration was evaluated. Recovery time was comparable between Dex and placebo, benzodiazepines, and opioids [SMD = 0.15, 95% CI (−0.08, 0.37), I^2^ = 68%, P = 0.20] [SMD = 0.10, 95% CI (−0.08, 0.28), I^2^ = 61%, P = 0.28] [SMD = −0.19, 95% CI (−0.42, 0.04), I^2^ = 38%, P = 0.10] (Figure 5).

#### 3.3.4 Perioperative complications

**FIGURE 6:**
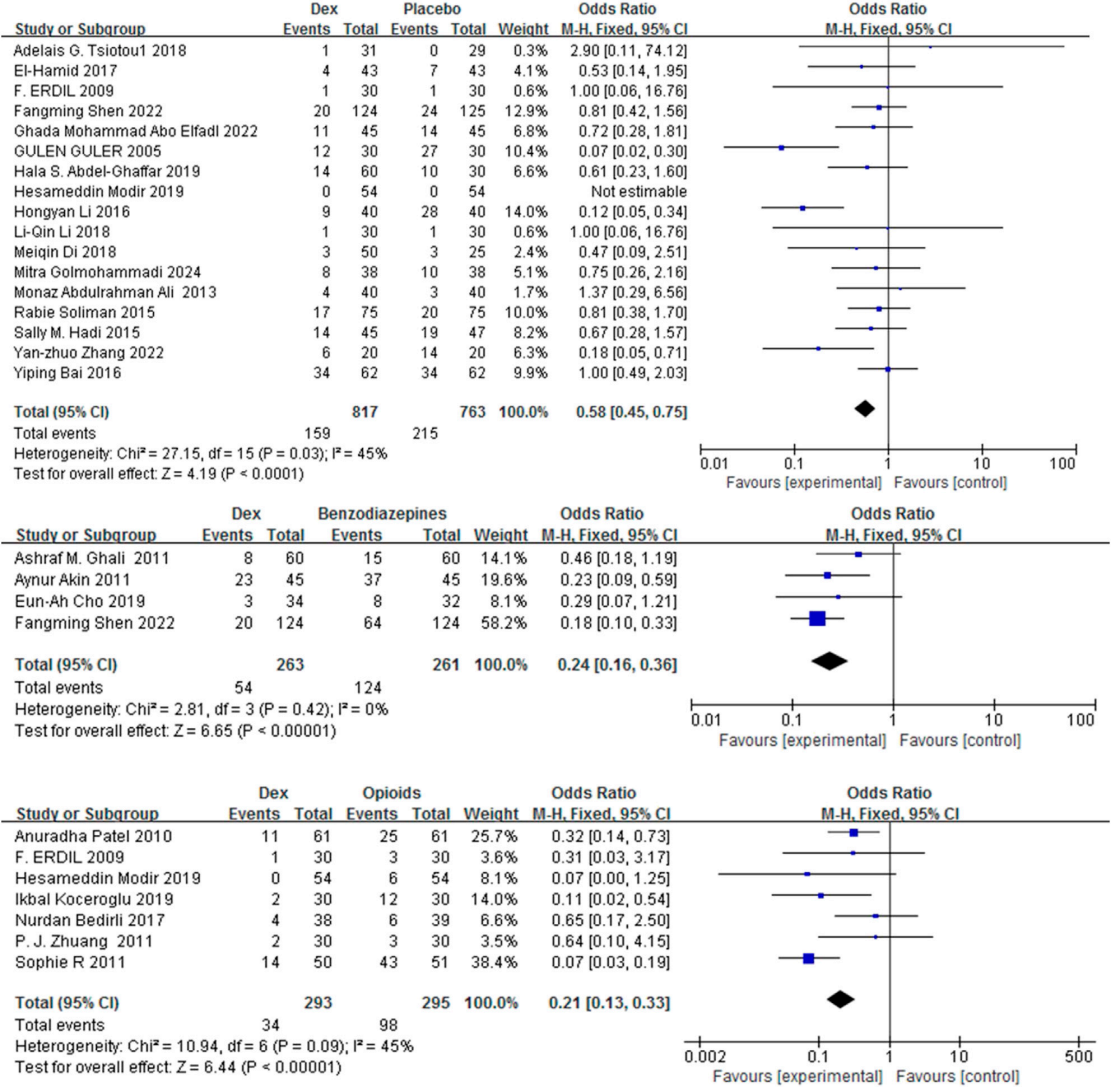
Occurrence of perioperative complications in Dex versus the control groups.

Among the 36 RCTs, 24 studies (Golmohammadi et al., 2024; Shen et al., 2022; Ali and Abdellatif, 2013; Tsiotou et al., 2018; Soliman and Alshehri, 2015; Bai et al., 2016; Li H. et al., 2018; El-Hamid and Yassin, 2017; Li L-Q. et al., 2018; Abo Elfadl et al., 2022; Guler et al., 2005; Abdel-Ghaffar et al., 2019; Di et al., 2018; Hadi et al., 2015; Zhang et al., 2022; Cho et al., 2019; Mahfouz et al., 2011; Akin et al., 2012; Zhuang et al., 2011; Bedirli et al., 2017a; Modir et al., 2024; Patel et al., 2010; Pestieau et al., 2011b; Erdil et al., 2009) including 2,294 children were analyzed. Compared with placebo, benzodiazepines, and opioids, Dex markedly reduced the incidence of perioperative complications [OR = 0.58, 95% CI (0.45, 0.75), I^2^ = 45%] [OR = 0.24, 95% CI (0.16, 0.36), I^2^ = 0%] [OR = 0.21, 95% CI (0.13, 0.33), I^2^ = 45%] (P < 0.0001) (Figure 6).

##### 3.3.4.1 Occurrence of perioperative complications

**FIGURE 7:**
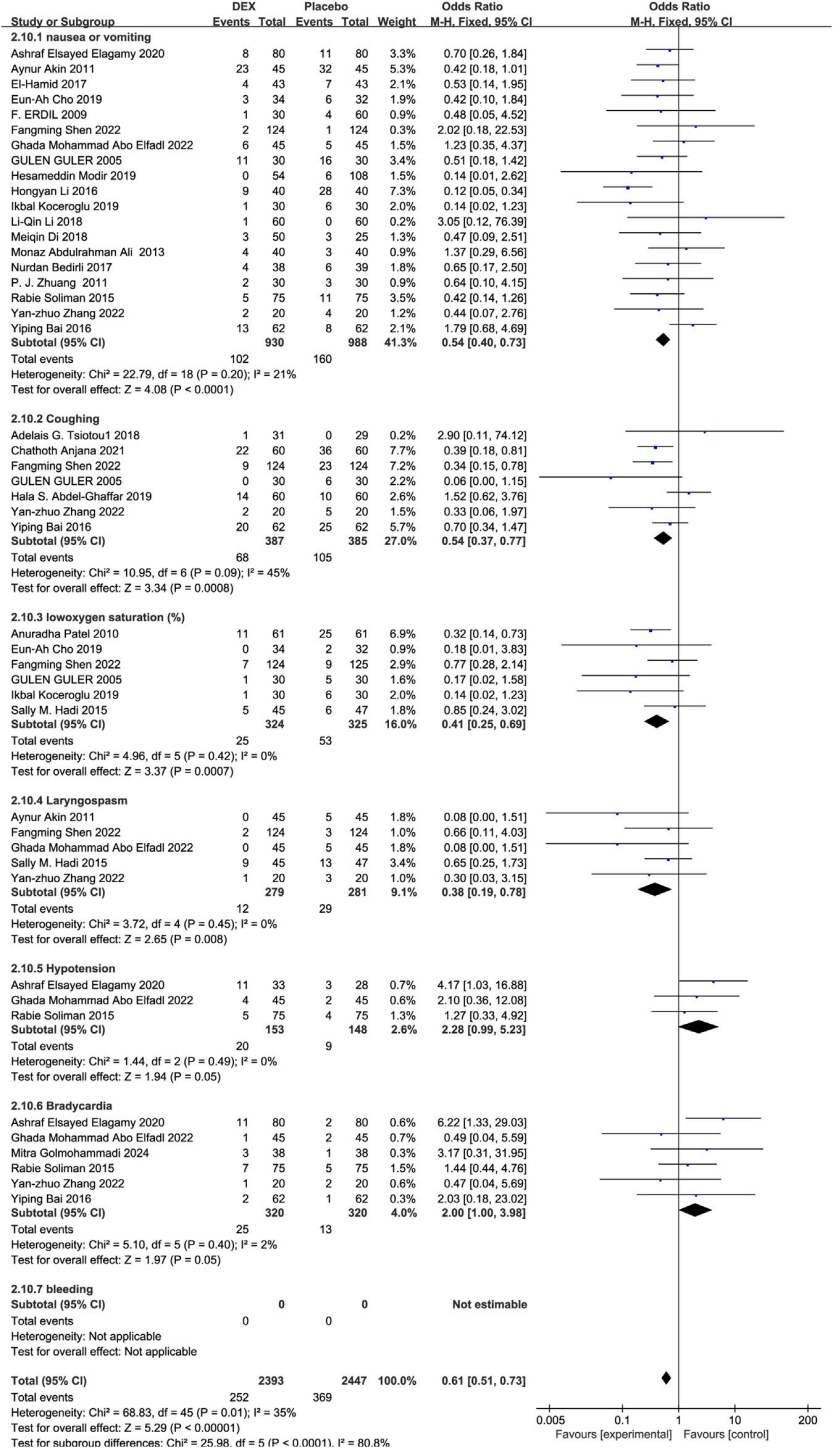
Perioperative complications associated with different administration types.

Dex reduced the risk of vomiting, cough, oxygen saturation (%), and laryngospasm compared with controls [OR = 0.54, 95% CI (0.40, 0.73), I^2^ = 21%,] [OR = 0.54, 95% CI (0.37, 0.77), I^2^ = 45%] [OR = 0.41, 95% CI (0.25, 0.69), I^2^ = 0%] [OR = 0.38, 95% CI (0.19, 0.78), I^2^ = 0%] (P < 0.05) (Figure 7). No significant difference was observed between the Dex and control groups regarding the risk of hypotension and bradycardia [OR = 2.28, 95% CI (0.99, 5.23), I^2^ = 0%, P = 0.05] [OR = 2.00, 95% CI (1.00, 3.98), I^2^ = 2%, P = 0.05] (Figure 7).

#### 3.3.5 Subgroup analyses

Guided by predefined hypotheses, subgroup analyses were performed to examine how Dex affects EA: stratifying studies by routes of administration (IV versus intranasal), timing of administration (post-anesthesia induction versus pre-surgery conclusion), and dosage variations [low (<0.5 μg/kg), moderate (≥0.5 to <1 μg/kg), and high doses (≥1 μg/kg)]. Table 2 presents the findings derived from subgroup analyses.

**TABLE 2:** Subgroup analysis results of the effect of Dex on the incidence of EA.

Subgroup outcomes	Number of studies	Results of heterogeneity test		Meta analysis results	
		P value	I^2^	OR (95% CI)	P value
(A) Different administration routes					
Intravenous	12	0.14	32%	0.27 (0.18,0.41)	<0.0001
Intranasal	3	0.03	71%	0.22 (0.18,0.38)	<0.0001
(B) Different administration time					
Post-anesthesia induction	7	0.12	41%	0.24 (0.17,0.35)	<0.0001
Pre-surgery conclusion	5	0.06	56%	0.34 (0.21,0.54)	<0.0001
(C) Different doses					
Low (<0.5 μg/kg)	2	0.22	34%	0.50 (0.24,1.04)	0.06
Moderate (≥0.5 to <1 μg/kg)	5	0.42	0%	0.23 (0.15,0.37)	<0.0001
High (≥1 μg/kg)	4	0.16	41%	0.17 (0.10,0.29)	<0.0001

In the subgroup analyses, Dex significantly decreased the frequency of EA, irrespective of whether it was administered via IV or intranasal routes [OR = 0.27, 95% CI (0.18,0.41), I^2^ = 32%] [OR = 0.22, 95% CI (0.18,0.38), I^2^ = 71%] (P < 0.0001) (Table 2). Timing of administration had consistent effects on both post-anesthesia induction and pre-surgery conclusion [OR = 0.24, 95% CI (0.17,0.35), I^2^ = 41%] [OR = 0.34, 95% CI (0.21,0.54), I^2^ = 56%] (P < 0.0001).

Furthermore, Dex markedly reduced EA at both moderate (≥0.5 to <1 μg/kg) and high doses (≥1 μg/kg) [OR = 0.23, 95% CI (0.15,0.37), I^2^ = 0%] [OR = 0.17, 95% CI (0.10,0.29), I^2^ = 41%] (P < 0.0001). In contrast, low-dose Dex (<0.5 μg/kg) did not significantly differ from the control [OR = 0.50, 95% CI (0.24, 1.04), I^2^ = 34%, P = 0.06].

## 4 Discussion

T&A in pediatric patients is a prevalent surgical procedure (Papp et al., 1998). Given its brevity, the anesthetics employed should demonstrate quick anesthesia induction, consistent anesthetic effects, minimal respiratory tract irritation, fast recovery, and a low incidence of complications (Thomsen and Gower, 2002; Kulka et al., 2001; D et al., 2010; Park et al., 2014). Consequently, the choice of a suitable anesthetic is crucial to mitigate complication risks and enhance the quality of anesthesia (Lodes, 1999; Maze and Tranquilli, 1991). Dex is recognized for its high selectivity toward α2-adrenoreceptors, enabling it to induce sedation, analgesia, and anxiolysis. It has a short half-life (1.8 h) and does not induce respiratory depression, which has supported its widespread use in several therapeutic contexts (Zhu et al., 2015). Owing to its dual analgesic and sedative properties, dexmedetomidine can serve as a viable adjunct or alternative agent for perioperative management in children undergoing T&A. Certain studies indicate that the prudent application of Dex and multimodal analgesia may lead to decreased opioid consumption or possibly its avoidance (Mann et al., 2021; Franz et al., 2019; Adler et al., 2021; Shi et al., 2019; Zhang et al., 2019; Sun et al., 2014). Consequently, an essential aspect in analyzing these results is the extent to which pain and agitation may be clinically intertwined.

This study demonstrates that, compared with placebo, benzodiazepines, and opioids, Dex was more effective in lowering the incidence of EA (Figure 3). This meta-analysis is the first to perform a specific subgroup analysis on the efficacy of Dex in preventing EA, providing novel, granular evidence on its optimal use that was not available in previous pooled analyses. Moreover, other measures have been employed to evaluate EA, including the Pediatric Anesthesia Emergence Delirium (PAED) scale developed by Sikich and Lerman, as well as five scales validated by Cole et al. (He et al., 2013; Hauber et al., 2015), which are extensively utilized. We incorporated the PAED scale into our study, and the results indicate that Dex significantly decreased PAED scores at 15, 30, and 45 min post-administration (Supplementary Figure S1), corroborating prior findings that Dex decreases the frequency of EA.

Pain, while not the only cause of EA, is a significant etiological element, and alleviating pain is often regarded as a means to reduce the frequency of EA linked to general anesthesia (Sun et al., 2014; Bedirli et al., 2017b). This review highlights the use of acetaminophen, NSAIDs, and a single steroid dose in pediatric T&A anesthesia (Mann et al., 2021). Compared with placebo or benzodiazepines, Dex decreased the need for rescue analgesics, reinforcing the analgesic properties of Dex in mitigating EA (Figure 2). In comparison to opioids, Dex appeared to lower EA. However, this assessment was derived from the analysis of only two studies. The observed lack of significant difference in rescue analgesic use [OR = 0.57, 95% CI (0.14, 2.27), I^2^ = 91%, P = 0.42] suggests notable uncertainty surrounding the comparative pain control benefits of Dex versus opioids. Consequently, these findings warrant cautious interpretation, and further empirical evidence is needed for confirmation.

Furthermore, recovery time was comparable between the Dex group and the control group, indicating that Dex does not delay or increase recovery to discharge time in the PACU. Several factors might account for these results. First, patients who did not receive Dex utilized supplementary medications, including opioids, for EA management (Zhuang et al., 2011; Modir et al., 2024; Albornoz et al., 2024). Second, the short half-life (under 2 h) of administered Dex may also inhibit an extended recovery duration.

PRAEs are the most prevalent complications associated with pediatric anesthesia. In pediatric cases, airway trauma from surgery induces edema in the upper respiratory tract and adjacent tissues in children, thus leading to the retention of secretions in the airway, and significantly increasing the risk of PRAEs (Shen et al., 2022). A significant percentage of children who had tonsillectomies encounter PRAEs, with the incidence reaching 50%. Dex has demonstrated efficacy in decreasing the incidence of PRAEs in pediatric patients with congenital heart disease (Zhang et al., 2020; von Ungern-Sternberg et al., 2013; von Ungern-Sternberg et al., 2019); however, conclusive data from rigorous assessments on its preoperative use for T&A-related PRAEs are currently insufficient. Our findings indicate that the occurrence of oxygen desaturation and laryngospasm dramatically decreased with Dex administration (Figure 7). Multiple pathways may contribute to this advantageous effect. First, Dex may increase the anesthetic level, thereby dampening airway reflex activity (Najafi et al., 2016; Wang et al., 2014). Second, its immunomodulatory effects, demonstrated through decreased interleukin-6 and tumor necrosis factor–α levels, may reduce airway inflammation and sensitivity (Tang et al., 2015). Third, Dex may correlate with reduced coughing and desaturation by decreasing the need for analgesics, attributable to its opioid-sparing properties. These findings indicate that the opioid-sparing properties of Dex may be advantageous for high-risk T&A patients. Moreover, hypotension or bradycardia occurred at similar rates in the Dex and control groups. Dex is known to induce hypotension, which may occasionally be preceded strangely by hypertension. This effect can be alleviated by avoiding fast infusion and bolus dosing. In studies with strict protocol adherence, Dex—used at conservative doses and not delivered intravenously—demonstrated a safety profile similar to the control group concerning hypotension and bradycardia occurrence (Ebert et al., 2000). In addition, due to its risks of hypotension and bradycardia pharmacological effects, it should only be used by healthcare professionals in settings equipped with medical monitoring facilities. Additionally, patients receiving this infusion should be under continuous monitoring, and should be discharged after demonstrating recovery from anesthesia and meeting established discharge criteria.

Subgroup studies of EA incidence were conducted to discern variations in the effects of administration route, timing, and dose. Both administration strategies and time points improved the incidence of EA. Furthermore, our findings indicated that compared with high doses (Dex ≥1 μg/kg), moderate doses (Dex ≥0.5, <1 μg/kg) markedly decreased the incidence of EA. Despite the results of the subgroup analyses, compared with the control treatment, low-dose Dex (<0.5 μg/kg) failed to significantly reduce the incidence of EA. Dex has dose-dependent effects on analgesia and sedation; lower dosages are associated with lower sedative efficacy, leading to an increased incidence of EA, similar to prior findings (Zh et al., 2021).

This study has several limitations. The exclusive focus on RCTs, while methodologically rigorous, may omit insights from other study designs. Heterogeneity in Dex regimens, adjuvant therapies, and small subgroup samples may affect generalizability. Although funnel plots revealed no publication bias (Supplementary Figure S2), language bias is possible given the exclusion of non-English studies. Moreover, the majority of RCTs have documented only these monitoring indicators within the post-anesthesia care unit, leaving the analgesic impact and its implications on neurological features post-discharge unexamined. Well-designed RCTs are essential for determining both the analgesic benefits and the post-discharge neurocognitive risks of Dex, especially regarding mood and focus capacity.

Finally, systematic studies comparing different administration routes, dosing timings, and dose regimens of Dex are currently lacking. Therefore, optimal routes of administration, specific doses, or timing strategies for Dex cannot yet be determined, and further research is needed.

## 5 Conclusion

Our study revealed that compared with different targets, Dex significantly reduced the overall occurrence of EA and perioperative complications. Furthermore, recovery time was comparable between subjects in the Dex group and those in the control group, indicating that Dex does not delay awakening to discharge readiness in the PACU. The present meta-analysis demonstrated the protective effect of Dex on EA and perioperative complications. Dex could be a useful analgesic option for children undergoing tonsillectomy with or without adenoidectomy. However, additional studies are needed to confirm these findings. Furthermore, high-quality research with a standard definition for EA is needed to explore the optimal administration route, dosage, and timing of Dex in pediatric anesthesia. Well-designed RCTs are essential for determining both the analgesic benefits and the post-discharge neurocognitive risks of Dex, especially regarding mood and focus capacity. Finally, further research is needed to compare the effects of different Dex doses in T&A.

## Data Availability

The original contributions presented in the study are included in the article/Supplementary Material, further inquiries can be directed to the corresponding author.
