# Supplementary figures and images for: Evaluating dexmedetomidine in mitigating emergence agitation and perioperative complications in pediatric tonsillectomy and/or adenoidectomy: a systematic review and meta-analysis
Source: Front Pharmacol. 2025 Oct 29;16:1681936. doi: 10.3389/fphar.2025.1681936 (PMC12605027; doi:10.3389/fphar.2025.1681936)

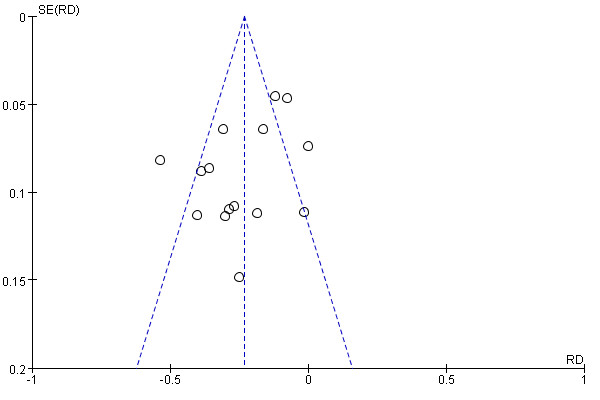

Supplement: Supplementary file 1 [file Image2.tif]

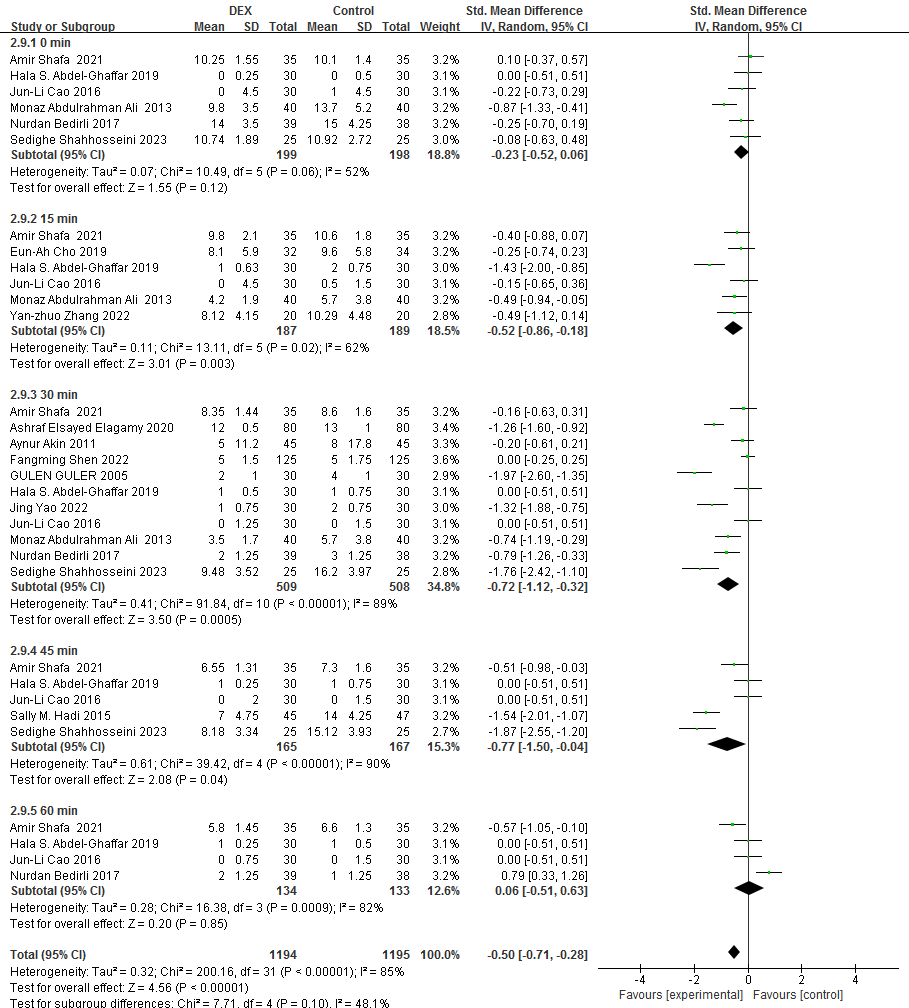

Supplement: Supplementary file 2 [file Image1.tif]
